# Comparative analysis of *emm *type pattern of Group A Streptococcus throat and skin isolates from India and their association with closely related SIC, a streptococcal virulence factor

**DOI:** 10.1186/1471-2180-8-150

**Published:** 2008-09-16

**Authors:** Vivek Sagar, Rajesh Kumar, Nirmal K Ganguly, Anuradha Chakraborti

**Affiliations:** 1 Department of Experimental Medicine & Biotechnology, Postgraduate Institute of Medical Education and Research (PGIMER), Chandigarh, India; 2 Department of Community Medicine, Postgraduate Institute of Medical Education and Research (PGIMER), Chandigarh, India; 3 Indian Council of Medical Research (ICMR), New Delhi, India

## Abstract

**Background:**

Group A streptococcus (GAS) causes a wide variety of life threatening diseases in humans and the incidence of such infections is high in developing countries like India. Although distribution of *emm *types of GAS in India has been described, there is a lack of data describing either the comparative distribution of *emm *types in throat versus skin isolates, or the distribution of certain virulence factors amongst these isolates. Therefore in the present study we have monitored the *emm *type pattern of Group A streptococcus throat and skin isolates from India. Additionally, the association of these isolates with closely related *sic *(*crs*), a multifunctional compliment binding virulence factor, was also explored.

**Results:**

Of the 94 (46 throat and 48 skin) isolates analyzed, 37 *emm *types were identified. The most frequently observed *emm *types were *emm*49 (8.5%) and *emm*112 (7.5%) followed by 6.5% each of *emm*1-2, *emm*75, *emm*77, and *emm*81. Out of 37 *emm *types, 27 have been previously reported and rest were isolated for the first time in the Indian Community. The predominant *emm *types of throat (*emm*49 and *emm*75) samples were different from those of skin (*emm*44, *emm*81 and *emm*112) samples. After screening all the 94 isolates, the *crs *gene was found in six *emm*1-2 (*crs*1-2) isolates, which was confirmed by DNA sequencing and expression analysis. Despite the polymorphic nature of *crs*, no intravariation was observed within *crs*1-2. However, insertions and deletions of highly variable sizes were noticed in comparison to CRS isolated from other *emm *types (*emm*1.0, *emm*57). CRS1-2 showed maximum homology with CRS57, but the genomic location of *crs*1-2 was found to be the same as that of *sic*1.0. Further, among *crs *positive isolates, *spe*A was only present in skin samples thus suggesting possible role of *spe*A in tissue tropism.

**Conclusion:**

Despite the diversity in *emm *type pattern of throat and skin isolates, no significant association between *emm *type and source of isolation was observed. The finding that the *crs *gene is highly conserved even in two different variants of *emm*1-2 GAS (*spe*A +ve and -ve) suggests a single allele of *crs *may be prevalent in the highly diverse throat and skin isolates of GAS in India.

## Background

Group A streptococcus (GAS, *Streptococcus pyogenes*) causes various diseases ranging from mild impetigo, pharyngitis and scarlet fever to more severe and serious sequelae such as rheumatic fever (RF), rheumatic heart disease (RHD) and acute glomerulonephritis [1]. The incidence of severe GAS diseases is high in children aged between 5–15 years and is more common in developing countries [2]. The prevalence of RF/RHD is known to vary from 0.3 to 5.4 children per 1000 in India [3].

Diversity in GAS strain is reflected not only among types of strains circulating in a particular community, but also the virulence factors associated with them [1]. GAS express a variety of virulence factors such as M protein, Streptolysin O and S, C5a peptidase, streptococcal pyrogenic exotoxins (Spe), streptococcal protective antigen (Spa) and streptococcal inhibitor of complement (SIC). Some of these virulence factors like Spa [4] or SIC [5] are found to be restricted in their distribution to specific *emm *types.

The complement binding protein, SIC, was first described by Akesson *et al*, 1996 [6] in M1 and later its variants were reported in M12, M55 and M57 [5,7]. Originally, SIC was characterized as an inhibitor of complement function that interferes with the function of the membrane attack complex by binding to one or more protein components associated with the complex. Subsequently SIC has also been shown to inhibit antimicrobial activity of lysozyme, secretory leukocyte proteinase inhibitor (SLPI), α and β-defensins, and Cathelicidin LL-37, which are components of the innate immune system [6,8,9].

The gene encoding SIC (*sic*) is highly polymorphic, both between different *emm *types and within strains of the same *emm *type [10]. Two forms of this gene have been identified, the closely related *sic *gene (*crs*) present in *emm*1 and *emm*57, and distantly related *sic *gene (*drs*) isolated from *emm*12 and *emm*55 [5]. Despite previous reports suggesting *crs *is associated with only *emm*1 and *emm*57 GAS isolates, Ma *et al *(2002) reported the association of the *crs *gene with other *emm *types including *emm*2, *emm*4, *emm*12, *emm*28, *emm*75, *emm*89, *emm*94 and *emm*112, leading to question about the distribution of *crs *[11].

Information regarding the circulating *emm *type is available from community screening [12] as well as from hospital data [13,14] in India. However, there is a lack of information regarding the distribution of *emm *types among strains isolated from different sites (throat and skin) and their virulence factors. Earlier, we have reported the presence of toxins [15] in GAS strains from our country and very recently, unraveled the conserved nature of other form of *sic *i.e. the *drs *gene [16]. Except for the presence of variable numbers of repeats, *drs *was found to be conserved not only within Indian isolates but also within the isolates from other countries. Here, we have explored for the first time the presence of the *crs *gene in *emm*1-2 isolates of Group A streptococcus from throat and skin infections. Although this study showed the conserved nature of the *crs*1-2 gene among Indian isolates, unlike *drs*, *crs*1-2 was found to be highly polymorphic when compared to isolates from other countries.

## Results and discussion

### Characterization of GAS strains by *emm *typing

For the first time, the *emm *type distribution of both throat and skin isolates of Group A streptococcus from India was studied and compared. 94 isolates associated with either throat or skin were categorized into 37 different *emm *types (Table 1). The majority of throat isolates were of *emm*49 and *emm*75, whereas *emm*44, *emm*81 and *emm*112 were mostly associated with skin infection. Only eleven *emm *types were found to be common in both throat and skin isolates. The distribution of *emm *types among throat and skin samples was different to the study of McGregor *et al *(2004) which involved samples belonging to geographic regions far away from Indian subcontinent [17]. However our data is quite similar to reports from other Asian countries, such as Japan [18] and Nepal[19].

**Table 1:** Distribution of different *emm *types among throat and skin isolates

*emm *type/subtype	Total Isolates	Throat Isolates	Skin Isolates
1–2.2	6	3	3
11.1	4	3	1
12.13	2	2	0
18.12	2	1	1
25.2	1	1	0
28.5	1	0	1
33.0	1	1*	0
43.3	2	2*	0
44.0	5	0	5
49.4	8	6+1*	1
55.0	1	0	1
67.0	1	1	0
68.0	2	2	0
69.1	3	3	0
70.0	1	0	1
71.0	1	0	1
74.0	1	1	0
75.0	6	5	1
77.0	6	4	2
80.0	2	1*	1
81.1	3	0	3
81.2	3	1	2
82.1	2	0	2
86.2	1	0	1
87.0	1	1	0
90-2	1	1	0
92.0	1	0	1
93.0	2	2*	0
100.1	1	0	1
102.2	1	0	1
103.0	2	0	2
104.0	3	0	3
106.0	1	0	1
112.2	7	1**	6
118.0	4	1	3
ST1731.1	1	0	1
ST2861 UK.1	1	0	1
NT	3	1+1*	1

* Represent strains Isolated from RF/RHD patients and ** Chorea patient NT: Represent nontypeable isolate.

The most frequently observed *emm *types (Table 1) among all isolates in this study were *emm*49 (8.5%), *emm*112 (7.4%) followed by 6.3% of *emm*1-2, *emm*75, *emm*77 and *emm*81. These most frequent *emm *types were not only different from previously reported most prevalent *emm *types in India [12-14] but also from epidemiological studies of isolates from other countries like Japan [18], Taiwan [20], Germany [21], Australia [22] and United States [23]. The difference in the most prevalent *emm *types of this study in comparison to earlier Indian reports may be due to the fact that the most prevalent serotypes within a population changes over time, which can be predicted by continuing surveillance [24]. Additionally, in this study both throat and skin isolates were studied, whereas earlier studies, involved throat samples only. Out of 37 *emm *types, 27 have been described in earlier reports from India, where as 16 were identical to *emm *types reported from Hong Kong [25] and interestingly, 29 to *emm *types of Ethiopia [26]. Identification for the first time of new *emm *types associated with skin infections in the Indian community further justifies the inclusion of skin isolates in this study.

On the basis of differences in amino acid sequence of the test strain from the parent strain in the type specific region of the *emm *gene, 37 types were subdivided in to 38 subtypes [27]. This observation is in contrast to the report available from Mexico where 31 types were differentiated into 66 subtypes [28]. In this study, out of 37 types only six isolates of *emm*81 could be divided into two subtypes i.e 81.1(3) and 81.2(3) (Table 1).

This study involved a small sampling of isolates from one area of a highly diverse country. The diversity in *emm *types reflect the extent of heterogeneity which exists among the strains prevalent in India. Only 11 *emm *types of this study correspond to *emm *types used in multivalent vaccine that is under trial in the USA [23]. This supports the development of a multivalent vaccine specific for this particular region covering all *emm *types prevalent in the Indian community. However development of such strategies needs further investigation with more samples belonging to each part of India.

### Screening of isolates for the *crs *(closely related *sic*) gene

**Figure 1:**
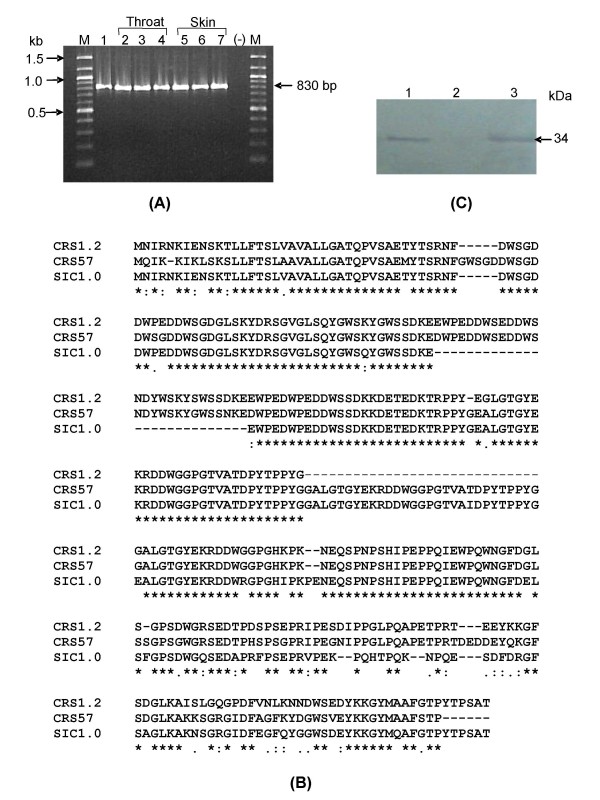
**Characterization of CRS**. (A) Screening of *crs *gene. Lanes: M, 100-bp Ladder (NEB, USA); 1, M1 reference strain used as positive control; 2–7, representative clinical *emm*1-2 isolates; (-), negative control (without template). B. Multiple sequence analysis of CRS1-2 from representative isolate, SIC1.0 (AP1 strain from Sweden) and CRS57 (reported from Australia). C. Western Blot analysis of CRS. Lanes: 1, M1 reference strain used as positive control; 2, negative control; 3, representative clinical *emm*1-2 isolate.

To elude the host defense and establish infection, GAS produces a number of virulence factors, including streptococcal inhibitor of complement (SIC). The polymorphic extra cellular complement binding protein SIC has not only pathological but also epidemiological significance. We have studied the *sic *gene distribution by screening ninety four GAS isolates. The six *emm*1-2 isolates (Table 1) were positive for the presence of the *crs *gene (*crs*1-2) specific ~830-bp fragment (Fig 1A), while in Japan the *sic *gene was isolated from 10 different *emm *types [11]. Therefore, the isolation rate (6.5%) of the *crs *gene in this study was found to be less compared to a study (77.3%) made in Japan [11]. Our data reports for the first time the presence of the *crs *gene from *emm *type 1–2, which is a distinct type, not a subtype of *emm*1.0.

### *crs *gene sequence and phylogenetic analysis

The *crs *gene product was further confirmed by sequence analysis using modified internal primers [29], which yielded a 912 bp full-length *crs *gene sequence (Accession number EF543156 – EF543161). The six *crs *positive isolates did not show intravariation either at the DNA or at the amino acid level. However, like *sic*1.0, *crs*1-2 also possessed a short repeat region (SRR), central long repeat region (LRR) and C proximal Proline rich region (PRR) as reported earlier [6]. A number of mutations such as insertions and deletions were observed in CRS1-2 throughout the sequence in comparison to SIC1.0 [6] and CRS57 [7]. Similar to SIC1.0, a deletion of five amino acids (GWSGD) was observed in CRS1-2 in comparison to CRS57 at position 40. However, an insertion of 27 amino acids (EWPEDDWSEDDWSNDYWSKYSWSSDKE) at position 82, similar to CRS57 in comparison to SIC1.0 has been noticed in CRS1-2. On the other hand a deletion of 29 amino acids (GALGTGYEKRDDWGGPGTVATDPYTPPYG) at position 165 makes CRS1-2 unique from both CRS1.0 and CRS57 (Fig 1B). Insertion and deletion sequences monitored in CRS1-2 in comparison to SIC1.0 and CRS57 is different from the 29 amino acid insertion (PPYGGALGTGYEKRDDWGGPGTVATDPYT) and the 31 amino acid deletion (GLSKYDRSGVGLSQYGWSQYGWSSDKEEWPE) sequence, most commonly observed in different alleles of *sic*1.0 [29]. It is likely, because this gene is under strong natural selection pressure [10], and as such harbors significant sequence variation and is highly divergent. The high number of allelic variations in *sic *is likely due to the fact that humans mount antibody response to SIC, a process that enhances variation by selecting escape mutants [30]. It is also reported that human anti-SIC antibodies are directed against virtually all regions of SIC that are highly polymorphic in natural population, which further strengthen the antibody mediated SIC diversification [31]. Phylogenetic analysis (Fig 1B) indicates the variant of SIC, CRS1-2, is more closely related to CRS57 [7] reported from Australia compare to SIC1.0 [6] reported from Sweden (Fig 1B). This correlation raises the possibility that *crs*57 may have originated from *emm*1-2 instead of *emm*1.0.

### Expression of *crs *gene at protein level

The secreted proteins from all *emm*1-2 isolates were seen in SDS-PAGE (12%) and specific antisera was used for western blot analysis which confirmed the expression of CRS1-2 from all these isolates. All six *emm*1-2 isolates (3 throat and 3 skin) showed the CRS specific protein of 34-KDa similar to *emm*1.0 (Fig 1C).

### Genomic location of *crs *1.2 gene

**Figure 2:**
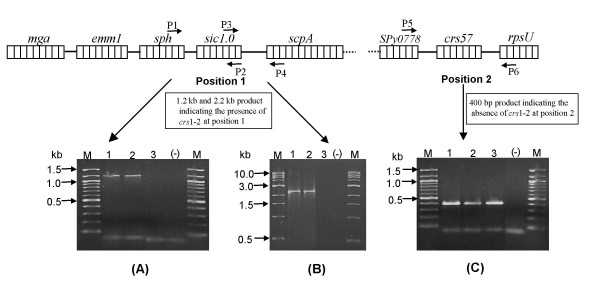
**Genomic location estimation of *crs*1-2**. (A) 1.2-kb product amplification by primer pair P1 – P2, Lanes: M, 100-bp Ladder (NEB, USA); 1, M1 reference strain used as positive control; 2, representative clinical *emm*1-2 isolate; 3, *sic *negative strain; (-), negative control (without template) (B) 2.2-kb product amplification by primer pair P3–P4, Lanes: M, 1-kb Ladder (NEB, USA); 1, M1 reference strain used as positive control; 2, representative clinical *emm*1-2 isolate; 3, *sic *negative strain; (-), negative control(without template) (C) 400-bp product amplification by primer pair P5 – P6, Lanes: M, 100-bp Ladder (NEB, USA); 1, M1 reference strain used as positive control; 2, representative clinical *emm*1-2 isolate; 3, *sic *negative strain; (-), negative control (without template).

In *emm*1.0, the *crs *gene is located within the *mga *regulon whereas it is located outside the *mga *regulon in *emm*57. In this study a PCR based method [6] was applied to find the genomic location of *crs*1-2. The amplified product of size 1.2-kb and 2.2-kb obtained by using primer pair P1 – P2 and P3 – P4 [6] respectively indicated the genomic location of *crs*1-2 to be the same as for *crs*1.0 (Fig 2A &2B). An additional PCR based analysis, which showed a 400-bp fragment by using primer pair P5 – P6 [7] further confirmed this observation (Fig 2C). Since *emm*1.0 and *emm*1-2 shares majority of alleles, as shown by MLST analysis [17], therefore such similarity in the genomic location of *crs*1-2 and *sic*1.0 is not unexpected.

### Screening of *sic *positive isolates for *spe*A

**Figure 3:**
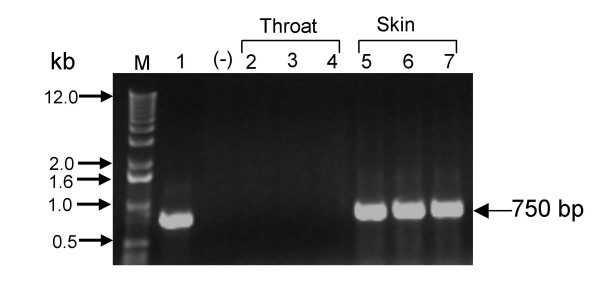
***spe *A gene amplification**. Lanes: M, 1-kb ladder (Invitrogen, USA); 1, M1 reference strain used as positive control; 2–7, representative clinical *emm*1-2 isolates; (-), negative control(without template).

To examine the presence of other virulence factors, *sic *positive isolates were screened for the phage encoded virulence factor *spe*A, which is also known as a source of diversity in these GAS strains [10]. Association of the *spe*A gene with *sic *positive isolates only from skin infection (Fig. 3) genetically differentiated them from throat isolates. This suggests the identification of two variants [10] of *sic *positive isolates, carrying the conserved *crs *gene. These two variants (*spe *A positive and negative), not only belong to different sources (throat and skin) but also to different regions of North India, and were also collected at different time periods.

## Conclusion

In the present study, we found that, although *emm *type pattern among throat and skin isolates was different but no significant association between *emm *type and source (throat and skin) was observed. Out of 37 different *emm *types, only six *emm*1-2 isolates were positive for *crs *gene validating its highly restricted distribution. Although no intravariation was observed in the *crs*1-2 gene, a large number of allelic variations were observed in the *crs*1-2 gene in comparison to *crs *genes reported from other countries. This suggests the *crs *gene is highly divergent in comparison to *drs*. Moreover, variation in virulence characteristics like possession of *spe*A in skin specific isolates not only differentiated *emm*1-2 isolates in two variants, but also reflects that virulence may be source specific, not type specific. The presence of conserved *sic *in these isolates further suggests a single allele of *crs *may be prevalent in the GAS isolates of Indian community, showing diverse *emm *type distribution in throat versus skin isolates.

## Methods

### Bacterial Strains

Group A streptococcus isolates (94) from throat (46 cases, comprising 36 from pharyngitis, nine RF/RHD and one Chorea) and Skin (48), were already available in the Department of Experimental Medicine and Biotechnology, Postgraduate Institute of Medical Education and Research, Chandigarh (India). Skin samples used in this study were collected during year 2000 – 2004 after obtaining ethical clearance from Institute ethics committee, Postgraduate Institute of Medical Education and Research, Chandigarh, from patients presenting with any suppurative skin lesion, wound, burn or rectum infection. However the throat isolates were collected from the throat of symptomatic patients (Pharyngitis, RF/RHD and Chorea) between the years 1995 and 2004. All these isolates were collected from hospital (Postgraduate Institute of Medical Education and Research, Chandigarh) as well as community screening (rural and urban slum) near Chandigarh after getting consent from parents.

### *emm *typing

*emm *gene sequencing was performed as previously reported [12]. DNA sequences were subjected to homology search against the bacterial DNA database . Pairwise comparison of the nucleotide homology for the first 160 bases of the hyper variable region of the *emm *gene was conducted to designate *emm *type to a particular strain. Types and subtypes were designated as described earlier .

### Identification and Sequence analysis of *crs*

Published primers [5] were used for the screening of the *sic *(*crs*) gene in GAS isolates. The complete *crs *gene was amplified and sequenced by using a different set of published primers [29]. A Specific internal primer was designed (ACCTAAGACCGAACAATCACCA) for *crs*1-2 sequencing. Sequencing was carried out in an Automated DNA sequencer, model number 310, Applied Biosystems, USA. Sequence data was compared with those already deposited in the Data bank by using clustal X program [32].

### Western blot analysis of CRS protein

GAS cultures were grown for 8 hrs and centrifuged at 10,000 × g for 10 mins. Supernatant proteins were precipitated with trichloroacetic acid (10% final concentration) at -20°C for approximately 20 mins. To retrieve the precipitated proteins the mixture was centrifuged at 16000 × g for 20 mins. The supernatant was discarded and the pellet was resuspended in 0.1 M NaOH. After running SDS-PAGE (12%), CRS protein was identified by using a specific antibody as described previously [7].

### *crs *gene location in genome

The location of the *crs *gene on the *mga *regulon was mapped by PCR using primer pairs (P1 – P2, P3 – P4 and P5 – P6) that were designed from sequence flanking the *crs *gene [6,7].

### Screening of *spe *A

*sic *positive isolates were further screened for the *spe *A gene as reported earlier [15].

## Abbreviations

SIC: Streptococcal inhibitor of complement; CRS: closely related SIC; DRS: distantly related SIC; RF/RHD: Rheumatic fever/rheumatic heart disease.

## Authors' contributions

All the authors have gone through the final manuscript. This work was a part of the Ph.D thesis of VS, done under supervision of AC (laboratory study) and RK (Field study). NKG has critically evaluated the manuscript.
